# Effectiveness of HIIT compared to moderate continuous training in improving vascular parameters in inactive adults

**DOI:** 10.1186/s12944-019-0981-z

**Published:** 2019-02-04

**Authors:** Robinson Ramírez-Vélez, Paula Andrea Hernández-Quiñones, Alejandra Tordecilla-Sanders, Cristian Álvarez, Rodrigo Ramírez-Campillo, Mikel Izquierdo, Jorge Enrique Correa-Bautista, Antonio Garcia-Hermoso, Ronald G. Garcia

**Affiliations:** 1 Centro de Estudios para la Medición de la Actividad Física “CEMA”. Escuela de Medicina y Ciencias de la Salud, Universidad del Rosario, Bogotá, DC Colombia; 2 Grupo GICAEDS. Programa de Cultura Física, Deporte y Recreación, Universidad Santo Tomás, Bogotá, DC Colombia; 3 Department of Physical Activity Sciences, Universidad de Los Lagos, Osorno, Chile; 4 Research Nucleus in Health, Physical Activity and Sports, Universidad de Los Lagos, Osorno, Chile; 5 Department of Health Sciences, Public University of Navarre, CIBER de Fragilidad y Envejecimiento Saludable (CB16/10/00315), Tudela, Navarre Spain; 6 Laboratorio de Ciencias de la Actividad Física, el Deporte y la Salud, Universidad de Santiago de Chile, USACH, Santiago, Chile; 7 Martinos Center for Biomedical Imaging, Department of Radiology, Massachusetts General Hospital, Harvard Medical School, Boston, MA USA; 8 Neurovascular Science Group, Fundación Cardiovascular de Colombia, Floridablanca, Santander Colombia; 9 Connors Center for Women’s Health and Gender Biology, Brigham and Women’s Hospital, Harvard Medical School, Boston, MA USA

**Keywords:** Aerobic exercise, Arterial stiffness, Cardiovascular disease prevention, Endothelial dysfunction, Sedentarism

## Abstract

**Background:**

Strong evidence shows that physical inactivity increases the risk of many adverse health conditions, including major non-communicable diseases, such as cardiovascular disease (CVD), metabolic syndrome, and breast and colon cancers, and shortens life expectancy. We aimed to determine the effects of moderate (MCT)- versus high-intensity interval training (HIT) on vascular function parameters in physically inactive adults. We hypothesized that individualized HIT prescription would improve the vascular function parameters more than the MCT in a greater proportion of individuals.

**Methods:**

Twenty-one inactive adults were randomly allocated to receive either MCT group (60–75% of their heart rate reserve, [HRR] or HIT group (4 min at 85–95% of peak HRR), 3 days a week for 12 weeks. Vascular function (brachial artery flow-mediated dilation, FMD [%], normalized brachial artery flow-mediated dilation, FMDn [%], aortic pulse wave velocity, PWV [m·s^− 1^], AIx, augmentation index: aortic and brachial [%]), were measured at baseline and over 12 weeks of training. In order for a participant to be considered a responder to improvements in vascular function parameters (FMDn and PWV), the typical error was calculated in a favorable direction.

**Results:**

FMD changed by − 1.0% (SE 2.1, *d* = 0.388) in the MCT group, and + 1.8% (SE 1.8, *d* = 0.699) in the HIT group (no significant difference between groups: 2.9% [95% CI, − 3.0 to 8.8]. PWV changed by + 0.1 m·s^− 1^ (SE 0.2, *d* = 0.087) in the MCT group but decreased by − 0.4 m·s^− 1^ in the HIT group (SE 0.2, *d* = 0.497), with significant difference between groups: − 0.4 [95% CI, − 0.2 to − 0.7]. There was not a significant difference in the prevalence of no-responder for FMD (%) between the MCT and HIT groups (66% versus 36%, *P* = 0.157). Regarding PWV (m·s^− 1^), an analysis showed that the prevalence of no-responder was 77% (7 cases) in the MCT group and 45% (5 cases) in the HIT group (*P* = 0.114).

**Conclusions:**

Under the conditions of the present study, both groups experienced changed in vascular function parameters. Compared to MCT group, HIT is more efficacious for improving FMD and decreasing PWV, in physically inactive adults.

**Trial registration:**

ClinicalTrials.gov NCT02738385 registered on 23 March 2016.

**Electronic supplementary material:**

The online version of this article (10.1186/s12944-019-0981-z) contains supplementary material, which is available to authorized users.

## Background

Strong evidence shows that physical inactivity (< 150 min/wk. of moderate-intensity activity or < 75 min/wk. of high -intensity activity) increases the risk of many adverse health conditions, including major non-communicable diseases, such as cardiovascular disease (CVD), metabolic syndrome, and breast and colon cancers, and shortens life expectancy [1, 2]. Physical inactivity has a deleterious effect that is comparable to smoking and obesity and is now recognized as the fourth leading risk factor for global mortality, accounting for 6% of all deaths [2].

Growing evidence suggests that exercise training improves vascular structure and nitric oxide bioavailability and reduces CVD risk factors; improvements in endothelial function may explain a large proportion of the risk reduction [3]. A number of factors appear to influence the acute effects of exercise on endothelial function, including sex, exercise intensity and duration, and the timing of post-exercise vascular function measurements [3]. Training protocols involving traditional moderate continuous training (MCT) and high-intensity training (HIT) can improve endothelial function [4, 5] a response largely mediated by acute elevations in blood flow and laminar shear stress during individual exercise bouts [6, 7]. In line with this, a growing body of evidence has demonstrated comparable or superior improvements in cardiovascular function using low-volume HIT compared to MCT [5].

Additionally, three sessions of 4 min of HIT per week (12 min/week) was sufficient to improve aortic reservoir pressure (an independent predictor of CVD), and thus may be a time-efficient exercise modality for reducing cardiovascular risk in individuals with metabolic syndrome [5, 8]. Furthermore, it was suggested that the ability of HIT to restore vascular homeostasis through the enhancement of shear stress-induced nitric oxide bioavailability may be another important mechanism that explains the protective role of exercise against non-communicable disease development [9]. Interestingly, despite this evidence, few randomized trials have directly evaluated the effects of sustained MCT or HIT on the cardiometabolic health of inactive adults [4, 9, 10].

There exists an inter-individual variability in vascular function, such that under the same stimulus, some subjects may achieve benefits, and are considered responders (Rs), whereas others may exhibit a worsened response or remain unchanged, and are considered non-responders (NRs) [11, 12]. Both genetic and environmental factors have been described to explain this previously reported phenomenon [13, 14]. However, all of these studies are primarily endurance or resistance training-based [9, 15] and most have not explored other exercise modalities such as HIT [16].

In Latin-American populations, information about optimal exercise timing for improving vascular function parameters is scarce. There is no consensus regarding optimal exercise timing for improving vascular function parameters. Additionally, determining the prevalence of “NRs” after an exercise program is relevant to optimize and predict responses in different populations (e.g., athletes or individuals with cardiometabolic risk factors).

The purpose of this secondary randomized clinical trial analysis was to compare the effects of MCT versus HIT on vascular function in physically inactive adult Latin-Americans. We hypothesized that individualized HIT prescription would improve the vascular function parameters more than the MCT in a greater proportion of individuals. Identifying the training regimen that has the most beneficial effects on each parameter could potentially lead to enhanced precision in prescribing exercise training intensity to achieve optimal outcomes in this population [16].

## Methods

### Sample and procedures

Details of the study design and methods of the primary HIT-Heart Study trial have been described elsewhere (ClinicalTrials.gov ID: NCT02738385) [17, 18]. Informed consent was obtained from each participant. The protocol was based on the Helsinki Declaration Accord (World Medical Association for Human Subjects). Moreover, ethical approval was obtained from the University of Santo Tomás (ID 27–0500-2015). Vascular function and fitness parameters were assessed at baseline and over 12 weeks of training. Briefly, the HIT-Heart Study conducted in 2013–2015 tested the efficacy of MCT versus HIT in changings biomarkers of endothelial and cardiovascular health (see Additional file 1: Figure S1 for CONSORT diagram).

Participants (*n* = 21) were recruited at the University of Rosario (Bogota, Colombia) from February 2015 to May 2016. Inclusion criteria were individuals aged 18–45 years who were inactive (< 150 min·wk.^− 1^ of moderate-intensity activity or 75 min·wk.^− 1^ of vigorous-intensity activity by applied a short version of the self-reported Global Physical Activity Questionnaire) and had a body mass index (BMI) ≥18 and ≤ 30 kg/m^2^. We excluded participants if they had a history of cardiovascular disease and related morbidities, diabetes mellitus 1 or 2, thyroid dysfunction, or cancer or if they were pregnant or smoked. All participants provided written informed consent before participating in the study. Participants were randomly assigned via a computer-generated, concealed, fixed block randomisation procedure to MCT (*n* = 10) or HIT (*n* = 11) groups. Data were obtained prior to randomisation by treating physiotherapists and physiologist, and then 12 weeks later by blinded assessors. Assessments were taken at baseline (Week 0) and 12 weeks after randomisation for all outcomes by experienced and blinded physiotherapists or exercise physiologist.

### Interventions

#### Moderate-continuous training (MCT) group

**Fig. 1:**
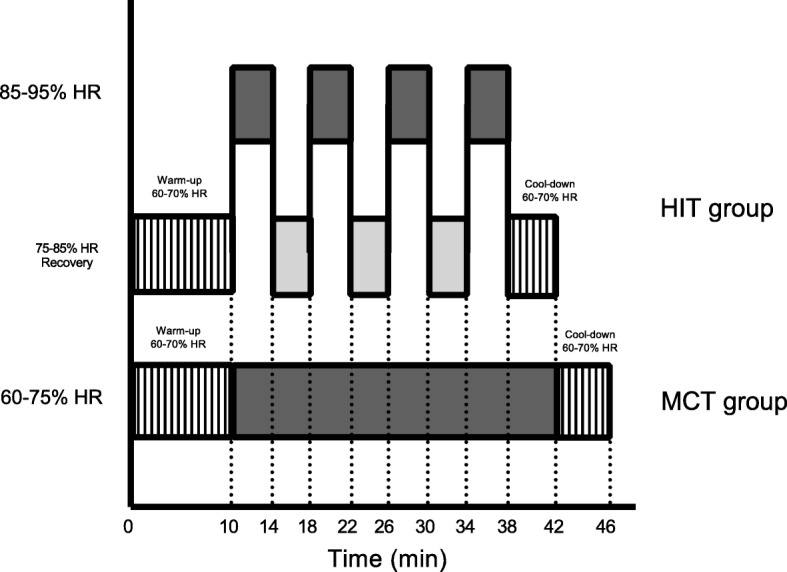
Schematic representation of a 4 × 4 HIT session (35-38 min) or MCT session (38-43 min)

The MCT protocol involved walking on a treadmill with the deck inclined to reach the desired intensity. Each preparatory period started with an exercise dose of 6 kcal·kg^− 1^·week^− 1^, which was increased progressively by 2 kcal·kg^− 1^·week^− 1^ until week 4 and was then maintained at 12 kcal·kg^− 1^·week^− 1^ for weeks 5 to 12. Exercise training sessions were designed to elicit a response in the acceptable moderate range, i.e., 60–75% of HRR and were adjusted according to ratings on the Borg scale [17, 18]. The rating of perceived exertion used was 12 to 15-point single-item scale ranging from 6 to 20 (6 “No exertion” and 20 “Maximum exertion”). Sessions consisted of a warm-up walk (10 min), followed by an aerobic exercise session (30-35 min) and a final relaxation/cool-down period (4 min). Exercise was performed in three sessions per week. During the supervised intervention, HR was recorded using a HR monitor (Polar Pacer, USA) to ensure compliance with the exercise stimulus at the predetermined target HR zone (Fig. 1).

#### High-intensity training (HIT) group

The HIT protocol involved fast walking and running on a treadmill with the deck inclined to reach the desired intensity. We calculated training energy expenditures according to participants’ age ranges associated with meeting the consensus public health recommendations from the Cardiometabolic HIT-RT Study [17, 18]. Each preparatory period started with an exercise dose of 6 kcal·kg^− 1^·week^− 1^, which was increased progressively by 2 kcal·kg^− 1^·week^− 1^ until week 4 and was then maintained at 12 kcal·kg^− 1^·week^− 1^ for weeks 5 to 12. The overall goal for the HIT group was to perform exercise sessions in 4 × 4 min intervals at 85–95% of HRR (with the target zone maintained for at least 2 min), interspersed with a 4-min recovery period at 75–85% of HRR. The speed and inclination of the treadmill were continuously adjusted to ensure that participants trained at the correct intensity. During each exercise session, participants adhered to the 12 -kcal·kg^− 1^·week^− 1^ energy expenditure format, which was equivalent to 300 kcal of energy expended by the end of the training and cool-down (4 min) periods, with a total exercise time ranging from 38 to 42 min. Exercise was performed in three sessions per week. During the supervised intervention, HR and Borg ratings were measured as described for the MCT group.

We selected 6 to 12 kcal·kg^− 1^·week^− 1^ per week because this dose of kcal·kg^− 1^·week^− 1^ has produced changes in VO_2_peak that placed approximately 70% of the initial sedentary population above the cut point for a low level of fitness [17, 18], as defined by both the American College of Sports Medicine (ACSM) [19] and the American Heart Association [20] guidelines for cardiovascular disease reduction.

The intensity to run/walk was related to a range of 85–95% (HIT) or 60–75% (MCT) of the maximum predicted heart rate according to the widely known equation (Karvonen), and the rest period was considered under a heart rate of 75–85% to HIT group of this marker. Thus, using the heart rate and oxygen consumption data obtained from the baseline fitness (cardiorespiratory uptake) test, the heart rate associated with an oxygen consumption of approximately 60% (MCT) and approximately 75–85% (HIT) were prescribed for each participant [19].

### Endothelial function arterial and wall parameters measures

The primary outcome measure was vascular function, as measured by flow mediated-dilation (FMD), aortic pulse wave velocity (PWV) and the augmentation index (Aix: aortic and brachial). FMD was measured as described in previous studies from our group [21] in the Colombian population using the protocol reported by Atkinson et al. [22]. A detailed description of the FMD technique can be found in a previous study^21^. FMD was expressed as % change = [(maximum − baseline diameter) / baseline diameter] × 100. Normalized brachial artery FMD (FMDn) was calculated according allometric relationship between Diameter (D) base and peak diameter (Dpeak), both measures in base-10 logarithm [22].

PWV was measured by analyzing the oscillometric pressure curves registered from the upper arm with arteriographic computer program (Arteriograph Software v.1.9.9.2; TensioMed, Budapest, Hungary). The algorithm measuring blood pressure in the arteriography device has been validated [23]. A detailed description of the PWV and AIx technique can be found in a previous study [21]. The reproducibility value as an estimate of the measurement errors for the repeat measurements between two sessions (*n* = 6) was low for the arteriograph (0.18 m·s^− 1^).

### Secondary outcomes

#### Anthropometric measurements

After completing another general information questionnaire, participants were instructed to wear shorts and a T-shirt to the physical exam. They were also required to remove all worn jewelry and metal objects. Once the subjects were barefoot and in their underwear, their body weight (kg) was measured using an electric scale (Model Tanita® BC-420®, Tokyo, Japan) with a range of 0–200 kg and with an accuracy of within 100 g. Height was measured with a portable stadiometer with a precision of 0.1 cm and a range of 0–2.5 m (Seca® 274, Hamburg, Germany). Body mass index (BMI) was calculated as the body weight in kilograms divided by the square of height in meters (kg/m^2^).

#### Cardiopulmonary exercise testing and training intensity

A maximal incremental test was performed by each participant on a treadmill (Precor TRM® 885, Italy) using a ramp protocol that simulates field running described by Ramírez-Vélez [18]. The criteria for exercise termination followed the American College of Sports Medicine recommendations [19], identified by an exercise physiologist who was present during each test. Maximum pulmonary oxygen uptake was defined as the mean cardiorespiratory uptake of the last 30s of exercise; the maximum HR was registered at the exercise peak.

Although diet was not controlled, participants met with the study’s dietician for nutritional assessment and counselling at baseline, and an individualized iso-energetic nutrition intervention plan was developed from the baseline food intake assessment according to participant preferences. This plan was standardized at 50–55% carbohydrates, 30–35% total fat, < 7% saturated fat and 15–22% protein, distributed across 3–4 meals per day [19].

Physical activity performed outside of the supervised exercise sessions (daily physical activity) was measured using Global Physical Activity Questionnaire for a 10 and 12 weeks [24]. MET-minutes/week were used to estimate the duration and intensity of physical activity during intervention.

### Sample size

The measurement of FMD, validated in several population studies, was selected as the critical variable to calculate the sample size [25, 26]. We determined the sample size for each group by power calculations using G*Power 3. A randomized clinical trial of the effect of aerobic training on FMD resulted in a standardized effect size (ES) of 0.3 to 0.6 for improvement in endothelial function [27]. An a priori power analysis estimated that a total sample size of 10 participants in each group. It was assumed that FMD would increase by approximately 1% over 12 weeks.

### Statistical analysis

All of the statistical analyses were performed using SPSS Version 25.0 (Chicago, IL, USA). Data were reported as mean, standard deviation (SD) or standard error (SE). Prior to the planned statistical analyses, a preliminary analysis was conducted (*Shapiro–Wilk tests*) to confirm the normality of the data. We used a generalized linear model (GLM) with repeated measures to analyze the influence of the different doses of exercise training on components of vascular function outcomes. Cohen’s *d* for effect size (ES) were also calculated to determine the magnitude of the group differences. ESs were classified as small, small-to-medium, and medium-to-large effects (< 0.20, 0.2–0.6 and 0.6–1.2, respectively) [28].

To classify the participants as “Rs” or “NRs” for improvements in FMD/PWV, the typical error (TE) was calculated, similar to the approach in our recent study [11, 13]. TE was calculated using the following equation: TE = SDdiff/√2, where SDdiff is the variance (standard deviation) of the difference in scores observed between the 2 repeats of each test. A “NR” was defined as an individual who failed to demonstrate a decrease or increase (whichever represented a beneficial change) that was greater than 2 times the TE. It was assumed that FMD would increase by approximately 0.9% and PWV reduce 0.5 m·s^− 1^, over 12 weeks. Chi- squared (X^2^) tests were used to assess the differences between the prevalence of “NRs” post-intervention for each group. All reported *P* values are two-sided (*P* < 0.05).

## Results

Additional file 1: Figure S1 shows the flowchart of this randomized clinical trial. A total of 28 physical inactive subjects were assessed for eligibility, of which seven were excluded for not meeting the inclusion criteria. Ten participants were randomly allocated to the MCT group, and 11 participants were randomly allocated to the HIT group. After allocation, one participant in the MCT group withdrew for reasons unrelated to this study (i.e., lack of time due to work schedule).

Table 1 presents the within- and between-group differences in vascular parameters following the training program. Peak brachial artery diameter significantly increased in the MCT group (+ 0.1 [SE 0.1] mm) and HIT group (+ 0.3 [SE 0.1] mm), with a medium-to-large effect (*d* = 0.474 to 0.732), with significant difference between groups: 0.1 mm (CI 95% = 0.0 to 0.3; *P* < 0.01), indicating positive adaptations following HIT compared with those following MCT. PWV changed by + 0.1 m·s^− 1^ (SE 0.2, *d* = 0.087) in the MCT group but decreased by − 0.4 m·s^− 1^ in the HIT group (SE 0.2, *d* = 0.497), with significant difference between groups: − 0.4 (95% CI, − 0.2 to − 0.7). There were no significant treatment effects on other vascular parameters.

**Table 1:** Anthropometric and vascular function parameters at baseline and changes after 12 weeks

	Groups				Within-Group Change Mean (SE)		Between-Group Difference in Change Mean (95% CI)	MCT effect *p* value (Effect size)	HIT effect *p* value (Effect size)
	Baseline Mean (SD)		After 12 weeks Mean (SD)						
	MCT (*n* = 10)	HIT (*n* = 11)	MCT (*n* = 9)	HIT (*n* = 11)	MCT (*n* = 9)	HIT (*n* = 11)			
Anthropometric									
Weight, kg	69.3 (15.3)	66.8 (10.9)	68.6 (13.5)	66.7 (10.5)	0.6 (0.6)	0.1 (0.4)	0.5 (−1.2 to 2.2)	0.341 (0.338)	0.717 (0.112)
Body mass index, kg/m^2^	23.6 (3.6)	25.5 (4.2)	23.4 (1.2)	24.4 (4.2)	0.2 (0.2)	1.1 (0.9)	−0.9 (−3.3 to 1.4)	0.393 (0.301)	0.274 (0.349)
Vascular function									
D_base_, mm	2.9 (0.5)	2.7 (0.4)	3.1 (0.4)	3.0 (0.4)	0.2 (0.1)	0.3 (0.1)	−0.1 (−0.2 to 0.1)	0.278 (0.388)	**0.043 (0.699)**
FMD, %	7.1 (3.2)	7.2 (5.6)	6.1 (6.5)	9.1 (5.2)	−1.0 (2.1)	1.8 (1.8)	2.9 (−3.0 to 8.8)	0.634 (0.165)	0.341 (0.301)
*D*_peak_ mm	3.1 (0.6)	2.9 (0.4)	3.3 (0.5)	3.3 (0.5)	0.1 (0.1)	0.3 (0.1)	**0.1 (0.0 to 0.3)**	0.193 (0.474)	**0.036 (0.732)**
*D*_diff_	0.2 (0.1)	0.1 (0.1)	0.2 (0.2)	0.2 (0.1)	0.0 (0.0)	0.1 (0.0)	0.1 (−0.1 to 0.3)	0.805 (0.085)	0.168 (0.449)
FMDn, %	6.5 (2.8)	7.4 (5.6)	4.9 (5.4)	8.1 (4.7)	−1.5 (1.9)	0.6 (1.6)	2.2 (−3.2 to 7.7)	0.451 (0.264)	0.693 (0.123)
PWV, m·s^−1^	6.7 (0.8)	7.1 (1.1)	6.8 (0.9)	6.7 (1.6)	−0.1 (-0.2)	0.4 (-0.2)	−**0.4 (**− **0.2 to** − **0.7)**	0.800 (0.087)	0.130 (0.497)
AIx (aortic), %	16.5 (5.2)	25.1 (16.5)	16.5 (8.4)	26.3 (14.6)	0.0 (2.7)	1.1 (2.1)	−1.1 (−3.5 to 1.3)	0.991 (0.004)	0.617 (0.156)
AIx (brachial), %	−41.7 (10.4)	−24.5 (32.7)	− 41.7 (16.5)	−22.3 (28.9)	0.0 (5.4)	−2.2 (4.2)	−2.2 (− 6.8 to 2.4)	0.998 (0.001)	0.619 (0.155)

Data are presented as mean and standard deviation (SD) or standard error (SE). D, Diameter; FMD, Flow-mediated vasodilation; FMDn, Normalized flow-mediated vasodilation; PWV, Pulse wave velocity; AIx, Augmentation index. Bold values denotes significant differences at level *p < 0.05*

**Fig. 2:**
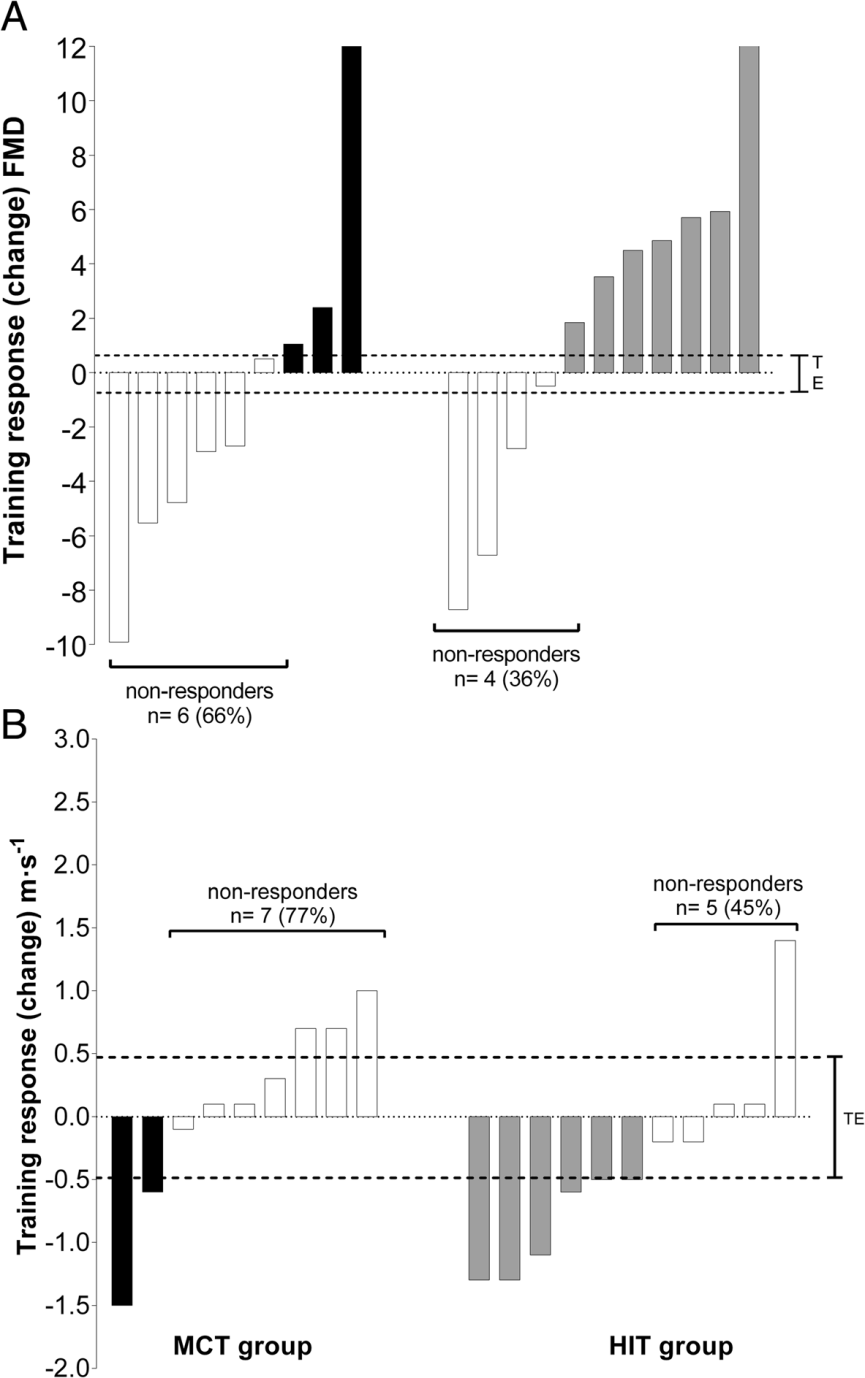
Differences in the prevalence of non-responders in vascular parameters after 12 weeks training. **a** FMD (%), **b** PWV (m·s^−1^)

Figure 2a and b show the mean values for individual changes in FMD (%) and PWV in both groups. Regarding FMD (%), the analysis showed a “NR” prevalence of 66% (6 cases) in the MCT group and 36% (4 cases) in the HIT group (*p* = 0.157). There was no significant difference in the prevalence of “NRs” for PWV between the MCT and the HIT group (77% versus 45%, *p* = 0.114).

No adverse events were reported over the course of this investigation. All data related to adherence and self-reported physical activity levels are presented in Table 2. Compliance with the study intervention was adequate, with 32/36 (89%) of participants receiving supervised exercise training. As expected self-reported physical activity increased as a result of training (F [1.65, 135.03] = 4.37; *p* < 0.001). Pairwise comparison analyses showed that the participants sustained these levels of vigorous or moderate physical activity at the 12-weeks follow-up. Between 10 to 12-week, walking differences over time in both groups was MCT group 945 MET-minutes/week vs HIT group 514 MET-minutes/week, (*p* < 0.001), but this difference was evident from high-intensity physical activity levels (MCT group 885 MET-minutes/week vs HIT group 1168 MET-minutes/week, *p* < 0.001).

**Table 2:** Attendance to prescribed exercise sessions and self-reported physical activity

Variable	MCT (*n* = 9)	HIT (*n* = 11)	Group effect (P value)
Adherence (% of prescribed sessions completed), mean (SD)	98.7 (3.7)	98.4 (2.8)	0.969
Total number of sessions completed, mean (SD)	32.5 (1.3)	32.5 (0.9)	0.993
Total MET-minutes/week, mean (SD)	1100 (258)	1031 (147)	0.043
International Physical Activity Questionnaire (10 to 12 week)			
Walking MET-minutes/week, mean (SD)	945 (1890)	514 (1014)	< 0.001
Moderate MET-minutes/week, mean (SD)	200 (276)	128 (260)	< 0.001
Vigorous MET-minutes/week, mean (SD)	885 (712)	1168 (588)	< 0.001

*SD* standard deviation

## Discussion

To the best of our knowledge, this is the first randomized clinical trial studying the effects of exercise training intensity on vascular parameters and individual responses in physically inactive adults from a Latin-American population. These findings suggest that exercise training induces potent stimuli leading to improvements in vascular parameters (i.e., decrease in arterial wall thickness and increase in endothelial function). Compared to MCT group, HIT is more efficacious for improving FMD and decreasing PWV in physically inactive adults, suggesting the presence of different regulatory mechanisms and time courses for induction.

HIT and MCT on a treadmill have been previously shown to be highly effective in patients with metabolic diseases [29]. Additionally, exercise training has been shown to be an effective therapeutic strategy for vascular function improvement in different clinical populations [30]. A previous meta-analysis showed that HIT is more potent than MIT in enhancing FMD, with a mean difference of 2.2% [31]. Specifically, this review suggested that a 4 × 4 HIT protocol three times per week for at least 12 weeks is an effective form of exercise for enhancing vascular functions. Our study showed a mean difference of 2.9% in FMD between groups; however, the difference was not significant. Along the same line, our data indicate that while brachial artery diameter increased as a result of exercise, arterial function assessed by PWV (m·s^− 1^) decreased at high levels of exercise (Table 1).

It is conceivable that substantial and/or sustained increases in shear forces that occur during exercise bouts may be associated with improving FMD, because stimulation of vasodilation post-exercise may result in the inhibition of related biochemical pathways [30]. In line with this, a meta-analysis of prospective studies reported a 13% reduction in the risk of cardiovascular events with a 1% increase in FMD; therefore, the magnitude of change in FMD following HIT (pre- vs. post-HIT + 1.8%) was deemed to be clinically significant in our study (*d* = 0.301 _[small-to-medium effect size]_).

Differences in exercise and experimental protocols in our study may have also contributed to discrepancies in our findings; however, this hypothesis remains to be tested. Our study showed that exercise intensity influences FMD response; however, FMD following exercise was attenuated in the MCT group but enhanced in the HIT group. Siasos et al. [7] suggested that both acute HIT and MCT can favorably affect endothelial function in healthy young adults, indicating another cardioprotective effect of exercise preventing the progression of atherosclerosis. The effects of these intense exercise regimens on FMD reflect a combination of hemodynamic changes and endothelial nitric oxide-dependent mechanisms [4, 10]. Exercise induces increases in blood flow, and augmented blood flow causes vasodilation, which directly impacts the magnitude of FMD [22, 32].

Regarding arterial wall parameters, aerobic exercise seems to significantly improve arterial stiffness, and this effect is enhanced at higher intensities of aerobic exercise and in participants with greater baseline arterial stiffness [31, 33]. PWV is widely recognized as a direct marker of arterial stiffness^33^. AIx index (aortic and brachial), are being more frequently used in studies as parameters of wave reflection [34]. In addition, an increase in PWV is linked with increased rates of cardiovascular incidences related to increased left ventricular afterload and wasted left ventricular energy [31, 33]. In this study, PWV increase by + 0.1 m·s^− 1^ (SE 0.2, *d* = 0.087) in the MCT group, but decreased by − 0.4 m·s^− 1^ in the HIT group (SE 0.2, *d* = 0.497), with significant difference between groups: − 0.4 (95% CI, − 0.2 to − 0.7). A previous systematic review and meta-analysis of RCTs reported that every 1 m·s^− 1^ increase in PWV is associated with a 12–14% increase in the risk of cardiovascular events and a 13–15% increase in the risk of CVD mortality. On the other hand, it was reported that aerobic exercise reduced PWV by 0.63 m·s^− 1^, which may be translated into an 8% reduction in cardiovascular events and a 9% reduction in cardiovascular mortality. Furthermore, subgroup analyses suggested that there may be bigger effects on PWV and, consequently, on cardiovascular events and mortality of aerobic exercise in higher risk participants (with PWV ≥ 8 m/s at baseline) and with longer durations of aerobic exercise (> 10 weeks) [31, 33]. In this line, HIT protocols seems to have a greater effect on peripheral than on central indices of arterial stiffness [7], which could justify our findings. However, several discrepancies between findings could also be due to differences in exercise modes or durations of HIT intervals; Ramos et al. [8] and Sawyer et al. [15] suggest that metabolic responses to HIT vary depending on the duration of the work-rest intervals.

Evidence from systematic reviews and experimental studies has demonstrated a positive effects of various exercise modalities (aerobic, resistance and combined training) on endothelial functions [7, 29, 31], but there are controversies regarding the effects of HIT on indices pertaining to arterial stiffness and wave reflection [7, 31, 35]. The mechanism by which HIT significantly reduces PWV more than MCT does could be associated with reduced exposure of the vasculature to reactive oxygen species that are often observed during high-volume exercise [36]. It is also possible that the higher volume of exercise in the HIT group may have resulted in the requirement of longer time for PWV recovery from repeated HIT bouts, thereby providing a more accurate representation of the cumulative effect of exercise intervention. These results may help identify the vascular wall that is more responsive and, conversely, the wall that is more resistant to the arterial stiffness-lowering effects of HIT [35].

On the order hand, the phenomenon of “NR” has been explored on performance variables [1] using MCT [12], resistance training [37], or HIT [11] in different age groups such as children [13], adults [29], and older populations [38]. Regarding FMD (individual responses), our analysis showed an “NR” rate of 66% in the MCT group and 36% in the HIT group (*P* = 0.157). Regarding PWV (m·s^− 1^), an analysis showed that the prevalence of no-responder was 77% (7 cases) in the MCT group and 45% (5 cases) in the HIT group (*P* = 0.114). This information can be useful when there are more than one risk factor to improve in physically inactive populations, and this knowledge can be useful for choosing exercise interventions with low rates of “NR” and high rates of improvements in particular outcomes. The data from some studies support our conclusion that exercise intensity plays an important role in modulating adaptations in vascular functions in response to exercise [4, 31, 32]. In line with this, several previous studies have reported increases [39, 40] decreases [27] or lack of change [10] in FMD following different exercise protocols. Unfortunately, none of these studies on exercise interventions reported on the rate of “NR”. Although some misleading studies have claimed the lack of non-responders in 4-week training intervals^1^, more recently, this phenomenon has been confirmed after 6 weeks and 6–8-months of exercise by relevant authors in the field [1].

In any case, the term “NR” may be related more to semantics, as the authors demonstrate a lack of response in some of the chosen outcomes (e.g., VO_2_peak, lean body mass, muscle strength, health status, etc.) across participants. Even the authors of reports that refute the so-called ‘myth’ of exercise non-response might agree that the term “NR” depends solely on the chosen clinical outcomes and that a non-responder for one outcome may not be a non-responder in another outcome [1, 11]. As technology advances and our understanding of the mechanisms driving exercise responses improves, scientists can continue to narrow the focus on clinical outcomes that are critical for improving the health of an individual, and healthcare practitioners can thus recommend exercise regimens on an individual basis rather than broadly suggesting the same exercise regimens for everyone.

Regardless of the mechanisms, it has been suggested that HIT may impair endothelium-dependent vasodilation due to an increase in reactive oxygen species, resulting in a reduction in nitric oxide bioavailability. Additionally, responses in FMD are inversely proportional to baseline arterial diameter. Further studies are necessary to establish optimal exercise training interventions for improving vascular health assessed by measuring FMD. Additionally, differences between the effects of different exercise regimens could be due to variability in their ability to generate greater blood flow through vessels supplying oxygen to the working muscles, which could in turn promote greater shear stress-induced nitric oxide bioavailability [39] and induce favorable endothelial adaptations [40]. In this context, several biologically plausible mechanisms may be used to explain the effects of exercise on the modulation of endothelial functions and arterial stiffness. It is widely known that exercise has the potential to reduce oxidative stress by increasing the efficiency of the antioxidant system, eventually improving endothelial dysfunction [40]. The main physiological mechanisms involve the up-regulation of endothelial nitric oxide synthase activity, as demonstrated in cell culture, animal and human studies, with a subsequent reduction in the expression of nicotinamide adenine dinucleotide (phosphate) (NAD(P)H)-dependent oxidase and the stimulation of free radical-scavenging systems that affect the levels of copper/zinc-containing superoxide dismutase, extracellular superoxide dismutase, glutathione peroxidase and glutathione [37]. Other studies have examined indices of antioxidant capacity, oxidative stress, and nitric oxide bioavailability as possible sources of improved FMD or reduced PWV. Future examinations should consider assessing these indices to fill in the gaps in the literature in their specific population of interest.

We did not observe improvements in AIx (% aortic or % brachial) body weight, or BMI, with exercise training, and therefore not likely clinically relevant. However, these findings should not deter future investigations from examining these indices. It remains possible that a larger sample size, or a sample with more clinically relevant pre-training states, such as hypertension, obesity, and elevated PWV, may experience more dramatic reductions in these indices with MCT or HIT training.

The strengths of this study include the use of state-of-the-art measures of vascular functions with supervised exercise training in a non-clinical setting. In addition, adherence to the intervention was ≈89%. All subjects completed 32 of 36 exercise sessions, and research technicians supervised each session while HR was being monitored. A primary limitation of this study was the lack of a true control group without exercise. Thus, we are unable to determine causality in our interpretation of the observed exercise-induced improvements in cardiovascular health parameters. Furthermore, in studies comparing HIT and MCT that have included a control group (no-exercise), no changes in FMD were observed in the control group [29]. Due to this and other limitations (e.g., single site design), it is important to not over-interpret the results of this RCT [17]. Other limitations of this study include the lack of control over tobacco usage. Additionally, indices other than post-occlusion reactive hyperemia flux were not assessed in the present study. However, we cannot determine the directions of the associations nor any causality observed in this study with absolute certainty. Lastly, we did not measure plasma nitric oxide, anti-oxidants and cytokines; however, any additional information provided by these measures may help to explain our findings.

Identifying the training regimen that has the most beneficial effects on each parameter could potentially lead to enhanced precision in prescribing exercise training intensity to achieve optimal outcomes in this population. Under the conditions of the present study, physically inactive adults in both groups experienced changed in FMD. Not all vascular function measured responded the same to this type of exercise, suggesting different regulatory mechanisms and time courses for induction.

## Conclusion

This study demonstrates the efficacy of HIT in enhancing the cardioprotective effects of exercise on the progression of atherosclerosis in a physically inactive population. However, compared to MCT group, HIT is more efficacious for improving FMD and decreasing PWV, in physically inactive adults. Identifying the mechanisms of adaptation may help to optimize the exercise program to target these mechanisms.

## Additional file

Additional file 1: **Figure S1.** CONSORT guidelines flow diagram for enrolment and randomisation HIT-Heart Study. (TIF 265 kb)

## Abbreviations

AHA: American Heart Association
CEMA: Centre of studies in physical activity measurements (in Spanish)
CVD: Cardiovascular disease
FMD: Flow-mediated vasodilation
HIT: High Intensity interval training
HRR: Heart rate reserve
ICC: Intra-class correlation coefficients
MCT: Moderate intensity training
METs: Units of metabolic equivalence
NRs: Non-responders
PA: Physical activity
PWV: Aortic pulse wave velocity
Rs: Responders
